# Interpretable EEG Emotion Classification via CNN Model and Gradient-Weighted Class Activation Mapping

**DOI:** 10.3390/brainsci15080886

**Published:** 2025-08-20

**Authors:** Yuxuan Zhao, Linjing Cao, Yidao Ji, Bo Wang, Wei Wu

**Affiliations:** 1 Institute of Automation, Chinese Academy of Sciences, Beijing 100190, China; wangbo@ia.ac.cn (B.W.); wei.wu@ia.ac.cn (W.W.); 2 School of Software and Microelectronics, Peking University, Beijing 102600, China; 2201110751@stu.pku.edu.cn; 3 School of Mechanical Engineering, University of Science and Technology Beijing, Beijing 100083, China; yidaoji@ustb.edu.cn

**Keywords:** EEG, emotion recognition, convolutional neural network, gradient-weighted class activation mapping, interpretability

## Abstract

**Background/Objectives:** Electroencephalography (EEG)-based emotion recognition plays an important role in affective computing and brain–computer interface applications. However, existing methods often face the challenge of achieving high classification accuracy while maintaining physiological interpretability. **Methods:** In this study, we propose a convolutional neural network (CNN) model with a simple architecture for EEG-based emotion classification. The model achieves classification accuracies of 95.21% for low/high arousal, 94.59% for low/high valence, and 93.01% for quaternary classification tasks on the DEAP dataset. To further improve model interpretability and support practical applications, Gradient-weighted Class Activation Mapping (Grad-CAM) is employed to identify the EEG electrode regions that contribute most to the classification results. **Results:** The visualization reveals that electrodes located in the right prefrontal cortex and left parietal lobe are the most influential, which is consistent with findings from emotional lateralization theory. **Conclusions:** This provides a physiological basis for optimizing electrode placement in wearable EEG-based emotion recognition systems. The proposed method combines high classification performance with interpretability and provides guidance for the design of efficient and portable affective computing systems.

## 1. Introduction

Affective computing is an interdisciplinary field that integrates psychology, neuroscience, and artificial intelligence. Its goal is to enable machines to recognize, interpret, and respond to human emotions. This capability is pivotal for advancing human–computer interaction (HCI), particularly in applications such as mental health monitoring, adaptive learning systems, and personalized robotics.Electroencephalography (EEG)-based emotion recognition has garnered increasing attention due to its potential to provide objective and real-time emotional assessments. However, achieving high classification accuracy while maintaining physiological interpretability remains a significant challenge. Recent studies have explored various deep learning and machine learning methodologies to enhance EEG-based emotion recognition performance.

Sharma et al. [1] proposed an online emotion recognition system utilizing nonlinear higher-order statistics and deep learning algorithms. They employed discrete wavelet transform (DWT) to decompose EEG signals and extracted third-order cumulants (ToC) to analyze nonlinear dynamics. To optimize feature selection, particle swarm optimization (PSO) was applied, followed by a long short-term memory (LSTM) network for classification, achieving an 82.01% accuracy on the DEAP dataset.

Graph-based approaches have also been investigated in recent studies. Yin et al. [2] developed a novel deep learning model integrating graph convolutional neural networks (GCNN) and LSTM to capture spatiotemporal EEG features. Their model, trained on the DEAP dataset, achieved classification accuracies of 90.45% and 90.60% for valence and arousal, respectively, in subject-dependent experiments. Similarly, Gao et al. [3] proposed EEG-GCN, a self-adaptive graph convolutional network incorporating spatiotemporal attention mechanisms, which demonstrated superior performance in emotion recognition on SEED [4] and DEAP [5] datasets.

Deep learning-based methodologies have also shown promising results in enhancing feature extraction and classification performance. Cheng et al. [6] introduced R2G-STLT, a transformer-based approach utilizing hierarchical learning from electrode to brain-region levels. Their model effectively captured the spatiotemporal dependencies in EEG signals and outperformed state-of-the-art methods on multiple datasets. Wang et al. [7] explored a 2D convolutional neural network (CNN)-LSTM model, integrating a differential entropy feature matrix (DEFM) to preserve spatial correlations among EEG electrodes, achieving 91.92% and 92.31% accuracy for valence and arousal classification, respectively, on the DEAP dataset.

Several studies have focused on optimizing feature selection and dimensionality reduction to improve model interpretability and efficiency. Houssein et al. [8] proposed an enhanced Coati Optimization Algorithm (eCOA) for feature selection, which demonstrated significant improvements in classification accuracy for EEG-based emotion recognition. Celebi et al. [9] developed a hybrid deep learning framework combining empirical wavelet transform (EWT) with 3D CNN, BiLSTM, and gated recurrent unit (GRU) models, achieving competitive results on the DEAP dataset.Recent research has also explored multimodal approaches for emotion recognition. Zhao et al. [10,11] proposed a multiscale CNN-based model incorporating EEG and peripheral physiological signals, leveraging decision fusion techniques to enhance classification accuracy.

Despite these advancements, challenges remain in balancing model accuracy, interpretability, and computational efficiency. To address these issues, this paper proposes a convolutional neural network (CNN) model for EEG-based emotion recognition that achieves high classification performance with a lightweight structure. Furthermore, Gradient-weighted Class Activation Mapping (Grad-CAM) [12] is applied to enhance model interpretability by visualizing the contribution of individual EEG electrodes to classification decisions. This method reveals spatial patterns of emotion-related brain activity that are consistent with the emotional lateralization theory. Overall, our work contributes to the development of efficient, interpretable, and biologically grounded emotion recognition systems, providing guidance for future wearable EEG device designs.

The remainder of this paper is organized as follows: Section 2 describes the dataset, preprocessing procedures, and the architecture of the proposed convolutional neural network (CNN) model. Section 3 presents the experimental results, including classification accuracy, activation map visualizations, and a comparative analysis with existing models. Section 4 provides an in-depth discussion of the findings, the physiological interpretation and the broader implications. Section 5 concludes the paper and outlines future research directions.

## 2. Materials and Methods

### 2.1. Dataset

The DEAP [5] is an open dataset used by researchers to validate their models. It includes EEG signals from 32 channels and peripheral physiological data from 8 channels, recorded as 32 participants watched 40 one-minute videos. Each trial consists of 63 s of signals, with the first 3 s serving as baseline measurements, recorded in the absence of stimuli. After each video, participants rated their arousal, valence, liking, and dominance on a 1 to 9 scale. A preprocessed version is available, where the data was down-sampled from 512 Hz to 128 Hz, and a 4.0–45.0 Hz bandpass filter was applied. The EEG data size is structured as 32 (participants) × 40 (videos) × 32 (EEG channels) × 8064 (signals), including 384 baseline signals.

### 2.2. Preprocessing

A baseline signal processing approach initially developed by Yang et al. [13] has demonstrated significant efficacy in enhancing recognition performance. Their experimental results revealed that implementing this technique could boost classification accuracy by nearly 32% in affective computing applications. The methodology consists of three sequential operations. First, raw baseline recordings from all C channels are segmented into N fixed-length L segments, generating N matrices of C × L dimensions. Second, baseline references are computed by averaging across segmented data to produce a standardized C × L template matrix M. Finally, signal normalization is performed by subtracting template M from equally segmented target EEG data, producing optimized biological signals for downstream analysis. To gain deeper insight into the model’s decision-making process, we apply Grad-CAM to identify the most influential EEG channels (electrodes) that contribute to each classification, as described in the Results section.

### 2.3. Proposed CNN Model

**Figure 1:**
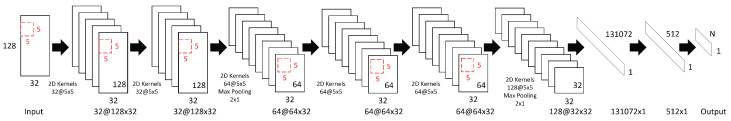
The architecture of the proposed convolutional neural network for EEG-based emotion recognition. Red rectangles represent 5 × 5 convolutional kernels. In the notation “X@Y × Z”, X denotes the number of filters, and Y × Z represents the output size (Y: temporal dimension, Z: number of EEG channels).

The architecture of the convolutional neural network model consists of six convolutional layers interleaved with two max-pooling layers, followed by two fully connected layers. The overall architecture of the network is illustrated in Figure 1, while detailed layer configurations, including output sizes, kernel sizes, number of filters (units), and stride parameters, are summarized in Table 1.

The input size is 128 × 32, where 128 corresponds to the number of consecutive time sample points processed at once, and 32 represents the number of EEG channels. The convolutional layers use a kernel size of 5 × 5 with zero-padding to preserve the spatial dimensions after convolution. The ReLU activation function is applied after each convolution operation to introduce nonlinearity. The choice of the 5 × 5 kernel size is motivated by the need to effectively capture both temporal and spatial patterns in EEG data. Compared to smaller kernels such as 3 × 3, the 5 × 5 kernels provide a larger receptive field per layer, enabling the network to better integrate information across adjacent time samples and EEG channels. This kernel size represents a balance between capturing sufficient context and maintaining computational efficiency.

Max-pooling layers with a pooling size and stride of 2 × 1 are used twice in the network—first after the third convolutional layer, reducing the temporal dimension from 128 to 64, and then after the sixth convolutional layer, further reducing it to 32. These pooling operations help reduce the data size along the temporal dimension and improve the robustness of extracted features.

The numbers of feature maps increase throughout the network as follows: 32 filters in the first and second convolutional layers; 64 in the third, fourth, and fifth convolutional layers; and 128 in the sixth convolutional layer. After the final max-pooling, the feature maps are flattened into a vector of size 131,072 before being passed to the fully connected layers. The first fully connected layer reduces this to a 512-dimensional feature vector, followed by the output layer with N units, corresponding to the number of classes in the classification task. The progressive increase in the number of filters from 32 in the initial convolutional layers to 128 in the deepest layer follows the principle of hierarchical feature extraction. Early layers with fewer filters focus on capturing fundamental, low-level features, while deeper layers employ more filters to model higher-level, more abstract representations. This design strategy balances feature richness with computational efficiency.

To effectively mitigate overfitting and improve generalization, several regularization techniques are incorporated in the model, outlined as follows: (1) Dropout layers with a keep probability of 0.5 are applied after the 1st, 3rd, 4th, and 6th convolutional layers as well as after the first fully connected layer. Dropout is implemented with structured noise shapes to randomly deactivate entire feature channels, which reduces co-adaptation of neurons and enhances model robustness. (2) Batch normalization is used after the 2nd, 4th, and 5th convolutional layers and after the first fully connected layer. Besides accelerating convergence during training, batch normalization serves as a regularizer that helps reduce overfitting. (3) The loss function incorporates L2 regularization on the network weights, constraining model complexity and preventing excessive fitting to the training data. (4) A learning rate decay strategy is employed, reducing the learning rate by 50% every 50 epochs, which helps the model converge more smoothly and avoid overfitting during later stages of training. (5) Residual connections are introduced between the 1st and 3rd convolutional layers and between the 4th and 6th convolutional layers to facilitate effective gradient flow and support better generalization in the deeper network. (6) Additionally, 10-fold cross-validation is used for all evaluations, and the network architecture is kept relatively lightweight to avoid excessive parameterization.

### 2.4. Model Interpretability via Grad-CAM

To enhance the interpretability of the proposed CNN model and to identify the spatiotemporal patterns most influential to its classification decisions, we employ the Gradient-weighted Class Activation Mapping (Grad-CAM) technique [14]. Grad-CAM is a post hoc visualization method that produces class-specific localization maps by leveraging the gradients of the target class score with respect to the feature maps of a selected convolutional layer. Unlike the original CAM method, Grad-CAM does not require architectural modifications such as replacing fully connected layers with global average pooling, and is applicable to a wide range of CNN architectures.

In our implementation, the final convolutional layer of the CNN is selected as the target layer for visualization. For a given input EEG trial and a target class *c*, we compute the gradient of the corresponding logit $y^{c}$ (before the softmax layer) with respect to the activations $A^{k}$ of each channel *k* in the target layer:

(1) $$\frac{\partial y^{c}}{\partial A^{k}}$$

These gradients are spatially averaged (global average pooling) over the full spatial extent of the feature map to obtain a weight $\alpha_{k}^{c}$ for each channel:

(2) $$\alpha_{k}^{c}=\frac{1}{Z}\sum_{i=1}^{H}\sum_{j=1}^{W}\frac{\partial y^{c}}{\partial A_{ij}^{k}},\;Z=H\times W$$

where *H* and *W* are the height and width of the feature map.

The Grad-CAM heatmap $L_{\mathrm{Grad}-\mathrm{CAM}}^{c}$ is then computed as a weighted combination of the feature maps, followed by a ReLU nonlinearity to retain only positive contributions, as follows:

(3) $$L_{\mathrm{Grad}\text{-}\mathrm{CAM}}^{c}=\mathrm{ReLU}\left(\sum_{k}\alpha_{k}^{c}A^{k}\right)$$

Finally, the resulting heatmap is upsampled via bilinear interpolation to match the original EEG input dimensions (128 × 32), enabling direct mapping to specific electrodes and time segments.

This procedure is applied separately for each target class in both binary and quaternary classification tasks. As an optional postprocessing step, the heatmaps can be averaged across trials or subjects to examine common activation patterns. In the Results section, these maps are analyzed for correspondence with established neurophysiological findings, such as hemispheric lateralization patterns in emotional processing.

## 3. Experimental Results

### 3.1. Classification Accuracy

We evaluate the proposed model using two EEG-based classification tasks: a binary task and a quaternary task. The model is constructed utilizing the TensorFlow framework [15] and runs on the NVIDIA Tesla K40c platform. For the training configuration, the learning rate is set at 1 × 10^−3^ in conjunction with the Adam Optimizer, the dropout retention probability is 0.5, and both the training and testing batch sizes are 240. A 10-fold cross-validation approach is adopted to gauge the model’s performance.

In the data preprocessing phase for a single trial signal (32 × 8064 dimensions), the baseline signal segment (32 × 384 dimensions) is first divided into three subsegments. These three baseline subsegments are used to calculate the mean value, generating a single 32 × 128 baseline mean. Additionally, the EEG data excluding the baseline is split into 60 subsegments (each 32 × 128 dimensions), and the previously computed baseline mean is subtracted from each of these subsegments. This process yields a preprocessed signal of 32 × 7680 dimensions.

The binary classification task includes the following two subtasks: low/high arousal (LA/HA) classification and low/high valence (LV/HV) classification. These subtasks are categorized according to arousal and valence values, using 5 as the threshold for distinction (≤5 for low, >5 for high). This threshold selection aligns with common practices in DEAP-based emotion recognition research to ensure comparability with existing literature [5,10,11]. Notably, retaining ratings of 4 and 5 in the low category (≤5) is critical for preserving sample size, as follows: excluding these middle ratings would reduce the number of samples by 40% for both binary subtasks (arousal: from 76,800 to 45,480 instances; valence: from 76,800 to 46,080 instances).

The quaternary classification task, in contrast, consists of the following four categories: low arousal low valence (LALV), high arousal low valence (HALV), low arousal high valence (LAHV), and high arousal high valence (HAHV), using the same threshold of 5 for arousal and valence. Excluding ratings of 4 and 5 would result in a more drastic 60% reduction in sample size (from 76,800 to 29,460 instances) and lead to substantial class imbalance. Therefore, these ratings are retained to ensure sufficient data volume and balanced class distribution.

The instance counts for each category in the DEAP dataset (with the current thresholding strategy) are detailed in Table 2.

The 10-fold cross-validation method is utilized to evaluate the model’s performance. The outcomes of each cross-validation iteration are detailed in Table 3, and the final result for each task is determined by taking the average accuracy across all 10 validation folds.

### 3.2. Visualization Using Grad-CAM

To illustrate the discriminative patterns learned by the proposed CNN model, we applied Grad-CAM to generate class-specific heatmaps for EEG trials. Specifically, we aim to identify which EEG channels (electrodes) and time segments are most influential in determining the output class. This enables us to gain insight into the underlying physiological patterns captured by the model and assess whether its decisions are based on meaningful neural activity.

**Figure 2:**
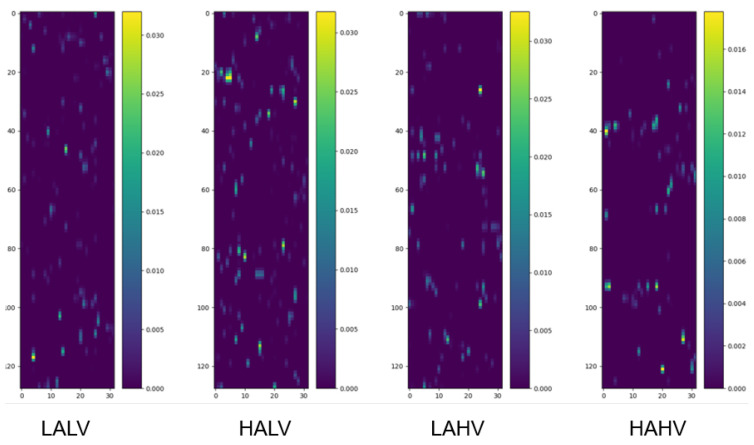
Class activation maps under quaternary classification task. Each map visualizes the spatiotemporal contribution of EEG channels to classification, as follows: The y-axis corresponds to 32 EEG channels, ordered according to the numbering convention adopted in the preprocessed DEAP dataset, which is based on the international 10–20 system for electrode placement, and the x-axis represents time segments (128 samples, corresponding to 1 s at 128 Hz). Warmer colors indicate regions (channel-time combinations) with greater influence on the model’s decision. Samples are randomly selected from the pooled dataset encompassing all participants, ensuring the observed patterns reflect generalizable channel contributions.

We randomly selected four samples from the pooled dataset (encompassing all participants) corresponding to the four categories under the quaternary classification task and drew their class activation maps, as shown in Figure 2.

**Figure 3:**
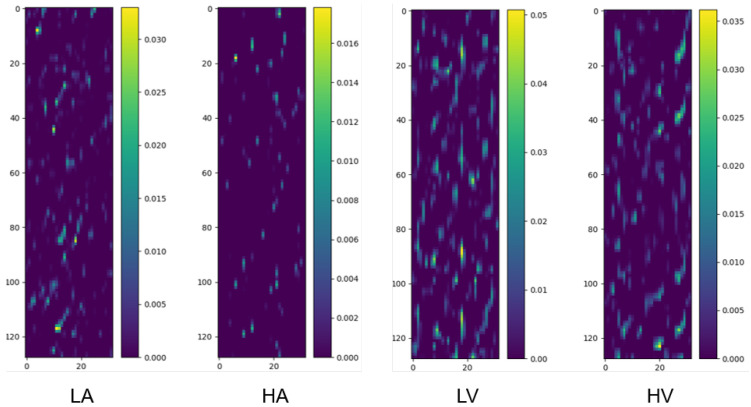
Class activation maps for binary classification tasks (Arousal: LA/HA; Valence: LV/HV). The y-axis corresponds to 32 EEG channels, ordered according to the numbering convention adopted in the preprocessed DEAP dataset, which is based on the international 10–20 system for electrode placement, and the x-axis represents 1 s time segments (128 samples at 128 Hz). Warmer colors denote regions with higher contribution to classification. Samples are randomly selected from the pooled dataset encompassing all participants, ensuring the observed patterns are not limited to specific individuals.

We randomly selected two samples from the pooled dataset (encompassing all participants) corresponding to the two categories under the binary classification task (Arousal and Valence, respectively) and drew their class activation maps, as shown in Figure 3.

The results of the class activation maps indicate that different channels have different contributions to the classification results, and the prediction of classification results does not rely on all channels. Therefore, we use category activation thermal maps to screen out channels that contribute significantly to the classification results, optimize the model, reduce the number of electrodes in the data acquisition process, reduce the difficulty of signal acquisition, and improve the comfort of the signal acquisition process.

**Figure 4:**
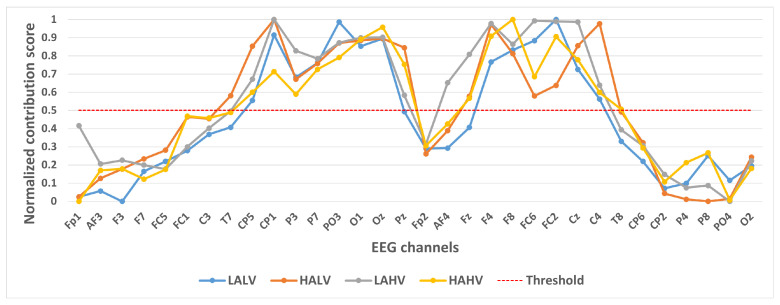
Contribution of different channels to classification results under quaternary classification task.

**Figure 5:**
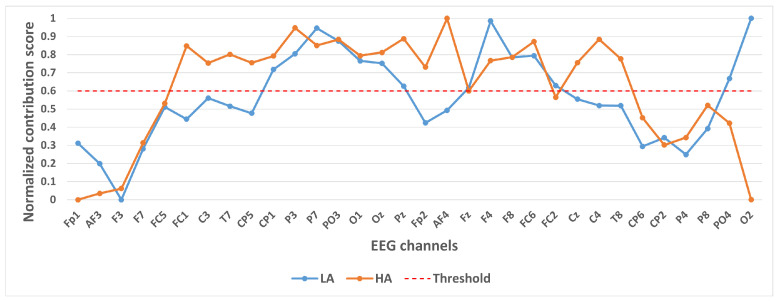
Contribution of different channels to classification results under binary classification task (Arousal).

**Figure 6:**
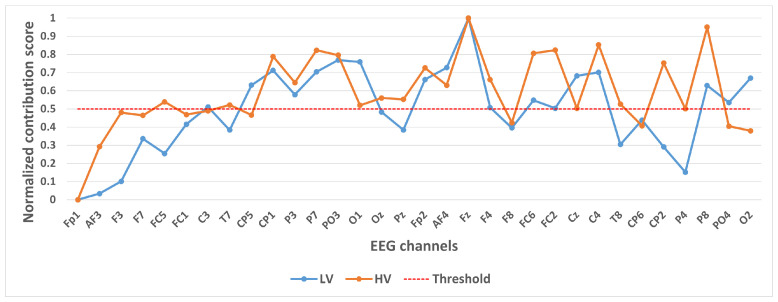
Contribution of different channels to classification results under binary classification task (Valence).

We took 14,000 samples under each category separately and plotted their category activation heat map. In order to analyze the contribution of different channels to the classification results, we take the mean value of each channel of the category activation heat map for each sample, and finally take the mean value of all the samples according to different categories and normalize them. These values were normalized to a 0–1 range to generate a Normalized Contribution Score, which reflects the relative importance of each channel in distinguishing emotional states. The results are shown in Figure 4, Figure 5 and Figure 6. The y-axis in these figures is labeled “Normalized Contribution Score”, where higher values indicate greater influence of the corresponding channel on the model’s classification decisions. This normalization ensures consistency across categories and tasks, facilitating the direct comparison of channel importance across different emotional classification scenarios.

In selecting electrodes with significant contributions, the determination of thresholds was based on the distribution characteristics of channel contribution scores and the principle of spatial continuity of brain regions. For the quaternary classification task, the contribution values of the 32 channels showed a natural boundary at 0.5 (most low-contribution electrodes had values < 0.5, while most high-contribution electrodes had values > 0.5). Using 0.5 as the threshold retained 13 electrodes, covering continuous brain regions in the frontal and parietal lobes, and avoided the disruption of spatial continuity caused by excessively high thresholds (e.g., 0.6). For the binary Valence task, the 14 electrodes selected by the 0.5 threshold formed a complete “prefrontal-parietal” collaborative network, whereas increasing the threshold to 0.6 would break the connectivity of this network. For the binary Arousal task, high-contribution electrodes were highly concentrated in the central-parietal cluster. A threshold of 0.6 precisely retained 10 core electrodes, excluded low-contribution redundant channels (e.g., marginal electrodes with contribution values < 0.6), and maintained the spatial continuity of electrodes within the cluster. The above threshold selection balances the retention of key information and optimization of electrode quantity, which is consistent with the neurobiological mechanisms of emotional processing.

Figure 4 shows the contribution of different channels to the classification results under the quaternary classification task. It is easy to notice that 13 channels, CP5, CP1, P3, P7, PO3, O1, Oz, F4, F8, FC6, FC2, Cz, and C4, contribute more to the classification.

Figure 5 shows the contribution of different channels to the classification results under the binary classification task (Arousal). It is easy to notice that the 10 channels CP1, P3, P7, PO3, O1, Oz, Pz, F4, F8, FC6 contribute more to the classification.

Figure 6 shows the contribution of different channels to the classification results under the binary classification task (Valence). It is easy to notice that 14 channels, CP1, P3, P7, PO3, O1, Fp2, AF4, Fz, F4, FC6, FC2, Cz, C4, P8, contribute more to the classification.

**Figure 7:**
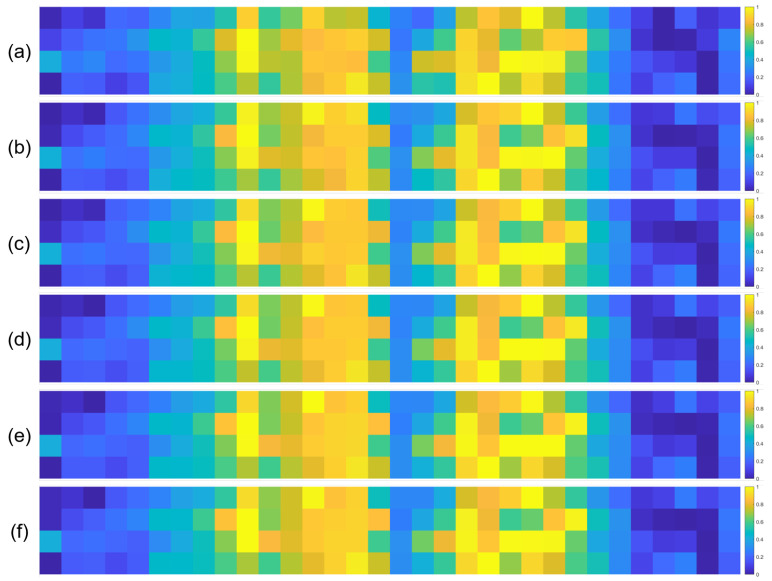
Heatmaps of normalized channel contribution values for six sample subset sizes under the quaternary classification task, where subfigures (**a**–**f**) correspond to 1000, 3000, 5000, 7000, 10,000, and 14,000 samples, respectively. In each subplot, the horizontal axis represents the 32 EEG channels, and the vertical axis represents the emotional categories. Warmer colors indicate higher relative contributions of the corresponding channel to classification.

**Figure 8:**
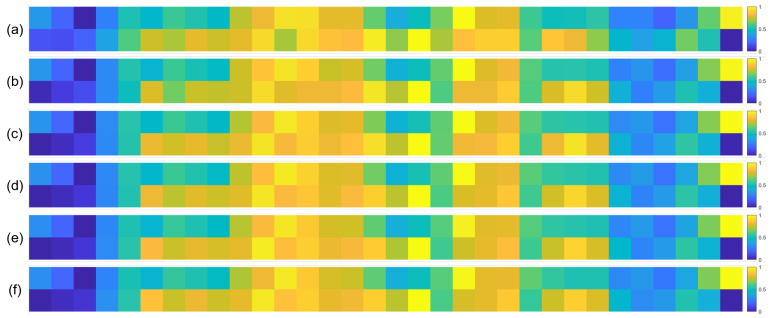
Heatmaps of normalized channel contribution values for six sample subset sizes under the binary classification task (Arousal), where subfigures (**a**–**f**) correspond to 1000, 3000, 5000, 7000, 10,000, and 14,000 samples, respectively. In each subplot, the horizontal axis represents the 32 EEG channels, and the vertical axis represents the emotional categories. Warmer colors indicate higher relative contributions of the corresponding channel to classification.

**Figure 9:**
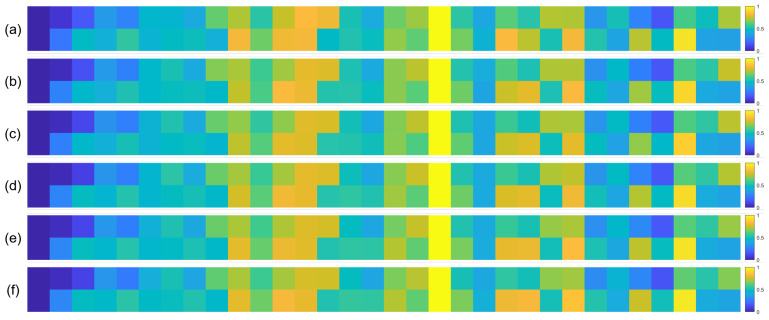
Heatmaps of normalized channel contribution values for six sample subset sizes under the binary classification task (Valence), where subfigures (**a**–**f**) correspond to 1000, 3000, 5000, 7000, 10,000, and 14,000 samples, respectively. In each subplot, the horizontal axis represents the 32 EEG channels, and the vertical axis represents the emotional categories. Warmer colors indicate higher relative contributions of the corresponding channel to classification.

To further validate the robustness of the identified important EEG channels across varying sample sizes, we conducted a systematic analysis by randomly extracting subsets of 1000, 3000, 5000, 7000, and 10,000 samples from the full dataset (14,000 samples). For each subset, we recalculated the normalized contribution scores of the 32 EEG channels and visualized their distribution through heatmaps, with separate analyses performed for the quaternary classification task (Figure 7), binary arousal classification (Figure 8), and binary valence classification (Figure 9).

A visual inspection of these heatmaps reveals striking consistency in the spatial patterns of channel contributions across all subset sizes. In each subplot, the warmer color clusters—indicating higher contribution—are concentrated in overlapping regions, regardless of sample size. For instance, in the quaternary classification task (Figure 7), the right frontal channels (F4, F8, FC6) and left parietal channels (CP5, P3, P7) consistently exhibit elevated contributions across all subsets, mirroring the activation pattern observed in the full 14,000-sample dataset. This visual coherence directly supports the stability of the model’s interpretive focus, as the key channels driving classification decisions remain unchanged even when the sample size is reduced by nearly 93% (from 14,000 to 1000).

To quantify this consistency, we performed statistical analyses by comparing the channel contribution vectors of each subset with those of the full dataset, using Pearson correlation coefficients (PCC) as the metric. The results, summarized in Table 4, Table 5 and Table 6, further confirm the robustness of our findings. In the quaternary task, all subsets achieved a PCC of 0.94 or higher, with most reaching a perfect correlation of 1.00, indicating near-identical contribution patterns. Similarly, the binary Arousal task yielded PCC values ranging from 0.87 to 1.00, while the valence task showed correlations between 0.81 and 1.00. These high correlation coefficients demonstrate that the relative importance of each channel is statistically consistent across different sample sizes.

Moreover, the tables explicitly list the high-contribution channels for each subset, revealing a remarkable overlap in the core set of electrodes. For example, in the valence classification task (Table 6), channels such as CP1, P3, P7, and F4 appear in all subsets, with only minor variations in peripheral channels (e.g., occasional inclusion or exclusion of Oz or Cz). This consistency in the core channel set reinforces the conclusion that the model’s interpretive insights are not artifacts of specific data partitions but reflect genuine neurophysiological markers associated with emotion recognition.

**Figure 10:**
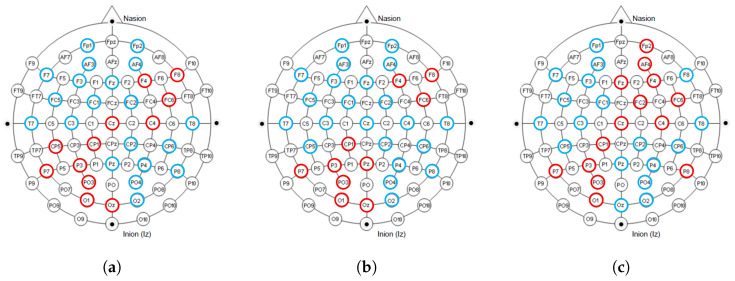
The distribution of electrodes that contribute significantly to classification under different tasks. The blue and red colors together mark the 32 electrode positions during the collection process in the international 10–20 system. The electrode positions that contribute significantly to classification are marked in red. (**a**) Quaternary classification task; (**b**) Binary classification task (Arousal); (**c**) Binary classification task (Valence).

The International 10–20 System is an internationally recognized method for describing and applying the position of scalp electrodes in electroencephalography or polysomnography. Figure 10 shows the electrodes used in the dataset collection process under the 10–20 system, and we mark the electrode positions that contribute significantly to the classification in red. It can be observed that in our proposed model, the electrodes that contribute significantly to classification are mainly distributed on the right side of the frontal lobe, the left side of the parietal lobe, and some intermediate regions. Furthermore, it showed similar results in all tasks. This result is consistent with biological discoveries [16,17].

### 3.3. Performance Comparison with Existing Models

For the two-class classification task, the proposed 2D-CNN model demonstrates superior performance, achieving 95.21% and 94.59% accuracy in arousal and valence classification, respectively. Table 7 presents a comparative analysis of our model against previous studies, primarily evaluated using 10-fold cross-validation on the DEAP dataset.

Compared to traditional machine learning approaches, such as the HOS+LSTM method proposed by Sharma et al. [1] and the EEG-GCN model by Gao et al. [3], our model outperforms them by approximately 10% and 13% in arousal classification and 10.43% and 12.82% in valence classification, respectively. Similarly, the deep learning-based methods from Yin et al. [2] and Cheng et al. [6], which incorporate graph convolutional and transformer-based architectures, achieve high classification performance, but our approach surpasses them by notable margins, with improvements of 9.94% in arousal and up to 14.33% in valence classification.

When compared with advanced deep learning architectures, such as the EWT-3D-CNN-BiLSTM-GRU-AT model by Celebi et al. [9] and the 2D-CNN-LSTM framework by Wang et al. [7], our method still achieves higher accuracy, improving arousal classification by 4.62% and 3.31%, and valence classification by 4.02% and 2.29%, respectively. While the CADD-DCCNN model from Li et al. [18] reaches competitive performance with 92.42% and 90.97% accuracy, our model still provides an advantage of approximately 2.79% and 3.62% in arousal and valence classification.

**Table 7:** The comparison of our model with previous studies in the binary classification task.

Research	Method	Accuracy		% Below Our Model		Year
		Arousal	Valence	Arousal	Valence	
Sharma et al. [1]	HOS+LSTM	85.21	84.16	10	10.43	2020
Yin et al. [2]	GCNN-LSTM	85.27	84.81	9.94	9.78	2021
Gao et al. [3]	EEG-GCN	81.95	81.77	13.26	12.82	2022
Ghosh et al. [19]	Gradient Ascent	88.67	86.95	6.54	7.64	2023
Cheng et al. [6]	R2G-STLT	86.22	80.26	8.99	14.33	2024
Houssein et al. [8]	HcF+eCOA+MLPNN	85.17	91.99	10.04	2.6	2024
Celebi et al. [9]	EWT-3D-CNN-BiLSTM-GRU-AT	90.59	90.57	4.62	4.02	2024
Wang et al. [7]	2D-CNN-LSTM	91.9	92.3	3.31	2.29	2024
Li et al. [18]	CADD-DCCNN	92.42	90.97	2.79	3.62	2024
**Our Model**	**2D-CNN**	**95.21**	**94.59**	**-**	**-**	**2025**

*Note:* HcF stands for hand-crafted features.

For the quaternary classification task, our 2D-CNN model achieves 93.01% accuracy on the DEAP dataset, outperforming previous approaches (Table 8). Compared to traditional methods like Gupta et al. [20] and Soroush et al. [21], our model improves accuracy by over 21% and 11%, respectively. It also surpasses deep learning models such as those of Sharma et al. [1] and Houssein et al. [8], with gains of up to 11% and 3.48%.

## 4. Discussion

In this study, we proposed a convolutional neural network (CNN) integrated with Grad-CAM for EEG-based emotion recognition. The goal is to enhance classification performance while addressing the challenge of interpretability in deep learning for affective computing. The model achieved high classification accuracies across multiple tasks as follows: 95.21% for arousal, 94.59% for valence, and 93.01% for the quaternary classification task, all of which outperform several state-of-the-art methods on the DEAP dataset.

The comparative results further validate the effectiveness of our model in both binary classification task and quaternary classification task. Across a range of baseline and advanced models—including traditional machine learning approaches (e.g., HOS+LSTM), graph-based deep learning models (e.g., EEG-GCN, GCNN-LSTM), and hybrid architectures such as EWT-3D-CNN-BiLSTM-GRU-AT—our 2D-CNN consistently achieved higher classification accuracies. These improvements, ranging from 2.3% to over 21% in some settings, suggest that our architecture strikes a favorable balance between model complexity and classification precision.

Unlike transformer-based or graph convolution methods that often involve heavy computational loads and require carefully structured inputs, our model maintains a relatively simple architecture while retaining strong generalization capability. This simplicity facilitates faster training, easier deployment, and better scalability, especially in resource-constrained environments. The inclusion of Grad-CAM also provides an interpretable mechanism absent in most prior studies, allowing for meaningful insights into spatial patterns of neural activation that support emotion recognition.

From a neurophysiological perspective, the Grad-CAM results align well with the theory of emotional lateralization, which suggests that the right frontal and left parietal cortices play crucial roles in emotional processing [16,17]. Our visualizations consistently identified these areas having greater influence on classification, across both binary and quaternary tasks. This not only reinforces the biological plausibility of our model but also provides a foundation for more interpretable machine learning in neuroscience applications.

Moreover, the identification of key electrode sites could inform the design of more efficient EEG acquisition systems. Reducing the number of channels while maintaining accuracy is critical for real-world deployment in wearable or mobile settings. This optimization can enhance the comfort and feasibility of wearable EEG systems in applications such as neurofeedback, brain–computer interfaces (BCIs), and emotion-aware assistive technologies.

Despite its strengths, the study has several limitations. First, while the Grad-CAM mechanism provides spatial interpretability, it does not yet offer insights into temporal dynamics, which are known to be important in emotional cognition. Future work may consider integrating temporal attention or recurrent architectures (e.g., BiLSTM or Temporal Convolutional Networks) to capture these dependencies. Additionally, although DEAP is a widely used benchmark dataset, generalization to other EEG datasets such as SEED [4], AMIGOS [24] or MAHNOB-HCI [25] should be explored to validate robustness. Finally, exploring subject-independent models would better reflect real-world usage scenarios, especially in adaptive systems where calibration time needs to be minimized.

## 5. Conclusions

This paper presents a CNN-based framework for EEG emotion recognition, augmented with Grad-CAM to improve the interpretability of deep models. Our approach achieved high classification accuracy on the DEAP dataset across both binary and multiclass emotion recognition tasks. Grad-CAM visualizations highlighted key brain regions—namely, the right prefrontal and left parietal areas—whose activations were found to be most indicative of emotional states. These results support the neurobiological plausibility of our model and affirm its effectiveness in extracting emotionally relevant features from EEG signals.

The main contributions of this study lie in the following: (1) integrating spatial interpretability into EEG-based affective computing models; (2) verifying the contribution of anatomically consistent brain regions; and (3) demonstrating a high-performance classification framework suitable for real-time applications.

In future work, we intend to expand the application of this technology to the domain of robotics. By integrating EEG-based emotion recognition with our previous research on theory of mind [26] and intention prediction [27], we aim to develop sophisticated affective human–robot interaction systems. This integration will enable robots to not only perceive and interpret human emotions with greater precision but also to adjust their behaviors in a way that demonstrates empathy and adaptability. Such systems hold the potential to substantially improve the quality of human–robot interactions, thereby enhancing their applicability and effectiveness across various real-world domains, including assistive robotics, service robots, and social companionship.To further enrich the emotional context and robustness of these systems, we will explore the integration of multimodal data—such as facial expressions, eye tracking, and galvanic skin response—alongside EEG signals. Moreover, adapting the model to run efficiently on low-power or embedded platforms will be essential for deploying emotion-aware technologies in real-world applications, including assistive robotics, mental health monitoring, and socially interactive systems.

## Figures and Tables

**Table 1:** Detailed layer configuration of the proposed CNN model.

Layer Type	Output Size	Kernel	Units	Stride
Input	128 × 32	\	\	\
First Convolutional Layer	128 × 32	(5, 5)	32	(1, 1)
Second Convolutional Layer	128 × 32	(5, 5)	32	(1, 1)
Third Convolutional Layer	128 × 32	(5, 5)	64	(1, 1)
First Max Pooling Layer	64 × 32	(2, 1)	\	(2, 1)
Fourth Convolutional Layer	64 × 32	(5, 5)	64	(1, 1)
Fifth Convolutional Layer	64 × 32	(5, 5)	64	(1, 1)
Sixth Convolutional Layer	64 × 32	(5, 5)	128	(1, 1)
Second Max Pooling Layer	32 × 32	(2, 1)	\	(2, 1)
Flatten Layer	131,072	\	\	\
First Fully Connected Layer	512	\	\	\
Second Fully Connected Layer	N	\	\	\

**Table 2:** Distribution of samples in the DEAP dataset.

**Binary Classification Task**				
	Arousal Categories		Valence Categories	
Label	Low (LA)	High (HA)	Low (LV)	High (HV)
Threshold	≤5	>5	≤5	>5
Sample Count	32,580	44,220	34,320	42,480
Total Samples	76,800		76,800	
**Quaternary Classification Task**				
Label	LALV	HALV	LAHV	HAHV
Arousal Threshold	≤5	>5	≤5	>5
Valence Threshold	≤5	≤5	>5	>5
Sample Count	16,440	17,880	16,140	26,340
Total Samples	76,800			

**Table 3:** Recognition accuracy (%) in the DEAP dataset.

Fold ID Mean & Std.Dev	Binary Classification Task		Quaternary Classification Task
	Arousal	Valence	
Fold 1	95.36	94.23	92.83
Fold 2	94.95	94.61	93.20
Fold 3	95.31	94.88	92.97
Fold 4	94.90	94.60	93.40
Fold 5	95.46	94.57	92.89
Fold 6	94.83	94.78	92.54
Fold 7	95.44	94.69	93.19
Fold 8	95.34	94.64	92.92
Fold 9	95.23	94.30	93.22
Fold 10	95.23	94.61	92.98
Mean	95.21	94.59	93.01
Standard Deviation	0.22	0.19	0.23

**Table 4:** Comparison of EEG channel contributions across different subset sizes under the quaternary classification task.

Quaternary Classification Task		
Subset Size	EEG Channels with High Contribution	PCC
1000	CP5, CP1, P3, P7, PO3, O1, Oz, F4, F8, FC6, FC2, Cz, C4	1.00
3000	CP5, CP1, P3, P7, PO3, O1, Oz, Pz, F4, F8, FC6, FC2, Cz, C4	0.94
5000	CP5, CP1, P3, P7, PO3, O1, Oz, F4, F8, FC6, FC2, Cz, C4	1.00
7000	CP5, CP1, P3, P7, PO3, O1, Oz, F4, F8, FC6, FC2, Cz, C4	1.00
10,000	CP5, CP1, P3, P7, PO3, O1, Oz, F4, F8, FC6, FC2, Cz, C4	1.00
14,000	CP5, CP1, P3, P7, PO3, O1, Oz, F4, F8, FC6, FC2, Cz, C4	1.00

*Note:* PCC (Pearson correlation coefficient) was computed between the EEG channel contribution vector obtained from the 14,000-sample subset and that from each smaller subset size.

**Table 5:** Comparison of EEG channel contributions across different subset sizes under the binary classification task (Arousal).

Binary Classification Task (Arousal)		
Subset Size	EEG Channels with High Contribution	PCC
1000	CP1, P3, P7, PO3, O1, Oz, Pz, Fz, F4, F8, FC6	0.93
3000	CP1, P3, P7, PO3, O1, Oz, Pz, Fz, F4, F8, FC6, FC2	0.87
5000	CP1, P3, P7, PO3, O1, Oz, Pz, Fz, F4, F8, FC6	0.93
7000	CP1, P3, P7, PO3, O1, Oz, Pz, Fz, F4, F8, FC6	0.93
10,000	CP1, P3, P7, PO3, O1, Oz, Pz, F4, F8, FC6	1.00
14,000	CP1, P3, P7, PO3, O1, Oz, Pz, F4, F8, FC6	1.00

*Note:* PCC (Pearson correlation coefficient) was computed between the EEG channel contribution vector obtained from the 14,000-sample subset and that from each smaller subset size.

**Table 6:** Comparison of EEG channel contributions across different subset sizes under the binary classification task (Valence).

Binary Classification Task (Valence)		
Subset Size	EEG Channels with High Contribution	PCC
1000	CP1, P3, P7, PO3, Oz, Fp2, AF4, Fz, F4, FC6, FC2, Cz, C4, P8	0.87
3000	CP1, P3, P7, PO3, O1, Oz, Fp2, AF4, Fz, F4, FC6, FC2, Cz, C4, P8	0.94
5000	CP1, P3, P7, PO3, O1, Oz, Fp2, AF4, Fz, F4, FC6, C4, P8	0.81
7000	CP1, P3, P7, PO3, O1, Fp2, AF4, Fz, F4, FC6, FC2, C4, P8	0.94
10,000	CP1, P3, P7, PO3, O1, Fp2, AF4, Fz, F4, FC6, FC2, Cz, C4, P8	1.00
14,000	CP1, P3, P7, PO3, O1, Fp2, AF4, Fz, F4, FC6, FC2, Cz, C4, P8	1.00

*Note:* PCC (Pearson correlation coefficient) was computed between the EEG channel contribution vector obtained from the 14,000-sample subset and that from each smaller subset size.

**Table 8:** The comparison of our model with previous studies in the quaternary classification task.

Research	Method	Accuracy	% Below Our Model	Year
Gupta et al. [20]	FAWT+RF	71.43	21.58	2019
Soroush et al. [21]	HcF+KNN+MSVM	81.67	11.34	2019
Sharma et al. [1]	HOS+LSTM	82.01	11	2020
Houssein et al. [8]	HcF+eCOA+MLPNN	89.53	3.48	2024
Goshvarpour et al. [22]	HcF+kNN+SVM	89.55	3.46	2024
Rakshe et al. [23]	HcF+CatBoost	84.5	8.51	2025
**Our Model**	**2D-CNN**	**93.01**	**-**	**2025**

*Note:* HcF stands for hand-crafted features.

## Data Availability

The original data presented in the study are openly available in the DEAP dataset repository at http://www.eecs.qmul.ac.uk/mmv/datasets/deap/ (accessed on 15 March 2024).
